# Support based on psychoeducation intervention to address quality of life and care burden among caregivers of patients with cancer: a randomized controlled trial

**DOI:** 10.3389/fpsyg.2025.1430371

**Published:** 2025-02-27

**Authors:** Seyedmohammad Mirhosseini, Fateme Imani Parsa, Hasan Moghadam-Roshtkhar, Mohammad Hasan Basirinezhad, Malihe Ameri, Hossein Ebrahimi

**Affiliations:** ^1^Department of Nursing, School of Nursing and Midwifery, Shahroud University of Medical Sciences, Shahroud, Iran; ^2^Student Research Committee, School of Nursing and Midwifery, Shahroud University of Medical Sciences, Shahroud, Iran; ^3^Imam Hossein Hospital, Shahroud University of Medical Sciences, Shahroud, Iran; ^4^Department of Epidemiology and Biostatistics, School of Public Health, Shahid Sadoughi University of Medical Sciences, Yazd, Iran; ^5^Center for Health Related Social and Behavioral Sciences Research, Shahroud University of Medical Sciences, Shahroud, Iran

**Keywords:** caregiver burden, quality of life, psychoeducation, cancer, caregiver

## Abstract

**Introduction:**

Cancer affects not only patients but also their family caregivers, causing increased caregiving burden and reduced quality of life. The aim of this study was to evaluate the impact of a psychoeducation intervention on improving the quality of life and reducing caregiving burden among caregivers of cancer patients.

**Methods:**

This study employed a non-blinded randomized controlled trial design involving 66 family caregivers of cancer patients undergoing chemotherapy in Shahroud, Iran in 2024. Of the 69 caregivers initially approached, one declined to participate, and two were excluded due to lack of smartphone access, leaving a final sample of 66 caregivers. Participants were assigned to either the psychoeducation intervention program or the control group using the quadruple block randomization method. The intervention spanned 3 months and consisted of six online group sessions lasting 35–45 min each. The psychoeducation intervention was delivered by trained psychiatric nurse. Data were collected before and 1 month after the intervention using the SF-36 quality of life questionnaire and the Novak and Guest care burden inventory. Statistical analysis was conducted using chi squared, independent t-tests, and the linear regression analysis with a significance level set at 0.05.

**Results:**

The primary outcome of this study was the change in caregivers’ quality of life and caregiver burden. Initially, both groups exhibited similar average scores for care burden and quality of life (*p* > 0.05). The intervention group showed a significant reduction in caregiving burden by 4.1 ± 13.7, whereas the control group experienced a slight increase of 2.5 ± 12.0. Similarly, quality of life scores improved by 4.7 ± 16.9 in the intervention group but declined by 8.6 ± 15.3 in the control group. Regression analysis indicated that the psychoeducation group demonstrated significantly lower caregiving burden scores and higher quality of life scores following the intervention compared to the control group.

**Conclusion:**

Caregivers of cancer patients often face significant burdens that impact their quality of life. Psychoeducational interventions focusing on coping, problem-solving, and stress management should be integrated into cancer care plans to provide essential support.

**Clinical trial registration:**

https://irct.behdasht.gov.ir/trial/54613, identifier IRCT20180728040617N3.

## 1 Introduction

Facing a cancer diagnosis, undergoing treatment, and managing the disease are stressful events. Patients with cancer, who are dealing with a chronic illness, are constantly exposed to various acute financial and non-financial distress (Lewandowska et al., 2021; Goyanka, 2021). The progression of the disease is accompanied by issues such as severe pain, increased fatigue, and psychological and emotional challenges, which can impact various aspects of these patients’ lives (Herschbach et al., 2020). In contemporary oncology, the focus extends beyond mere drug treatments to encompass a holistic understanding of patients’ and their families’ experiences. This approach prioritizes resource allocation, comprehensive care planning, and delivery that emphasizes quality of life and other subjective factors influencing care (Ho et al., 2018). Importantly, the burden of cancer is not borne solely by patients but also significantly affects their crucial family members, particularly their caregivers. Families of cancer patients, especially spouses, are greatly affected by the stress of this disease. In recent years, this impact has been defined as a dyadic coping phenomenon. This means that the disease not only affects the individual patients but also their family members (Turliuc et al., 2021). Dyadic coping is a systemic framework that describes how caregivers manage stressors, encompassing stress communication, individual strategies to support the other caregiver, and joint strategies to cope collaboratively (Falconier et al., 2015). Kayser et al. (2007) also introduced the term “We-disease,” highlighting that the burden of care should be considered for both the cancer patient and their family members (Kayser et al., 2007). These caregivers, often overlooked, provide financial, physical, emotional, and social support, assuming numerous responsibilities to meet patients’ needs (Kilic and Oz, 2019). It’s essential to recognize that the impact of cancer transcends patients, reaching their dedicated family caregivers. These individuals, informal and unpaid, share a personal connection with the patient, undertaking various physical, social, practical, and emotional tasks (Harding, 2013).

Family caregivers play a pivotal role in the modern healthcare system, providing support to patients both within and beyond medical facilities. In the context of cancer, family caregivers are tasked with assisting patients across various facets of their lives, ranging from aiding with basic daily activities to offering emotional, social, and financial assistance. This support extends throughout treatment, medication management, coordination of healthcare appointments, and facilitating patients’ daily routines (Dengsø et al., 2021; Adelman et al., 2014). Among the significant challenges faced by family caregivers in their caregiving role, caregiving burden stands out prominently. Caregiving burden refers to the perceived negative effects experienced while caring for a family member. This burden manifests in two primary forms: objective and subjective. Objective care burden entails the tangible tasks, energy expenditure, time commitment, and financial resources allocated to daily caregiving responsibilities. On the other hand, subjective care burden encompasses the emotional challenges encountered during caregiving, such as symptoms of depression, burnout, and stress (Caserta et al., 1996). Caregiving burden is recognized as a multidimensional construct, encompassing social, emotional, psychological, physical, and economic consequences for the caregiver (Vatter et al., 2018).

Caregivers of cancer patients face significant challenges in their personal lives, and assuming a caregiving role can intensify this burden. Moreover, many of these family caregivers lack formal training in caregiving and often find themselves responsible for medication administration, symptom management, as well as providing financial and emotional support (Fumaneeshoat and Ingviya, 2020). Research indicates that caregiving for cancer patients is more demanding compared to caregiving for individuals with other chronic illnesses, and caregivers providing intensive support due to imposed roles tend to experience poorer outcomes (Ochoa et al., 2020). High levels of caregiving burden are often associated with low levels of resilience among caregivers, placing them at greater risk for anxiety and depression. This heightened vulnerability may stem from increased financial strain due to treatment costs, loss of income, and social limitations. These factors collectively contribute to a decline in caregivers’ quality of life (Üzar-Özçetin and Dursun, 2020; Hastert et al., 2020).

As previously discussed, quality of life stands as a pivotal construct within the realm of caregivers for cancer patients. Quality of life is comprehensively defined as the amalgamation of perceptions, emotions, and thoughts that shape an individual’s evaluation of their own life. It encapsulates overall well-being, encompassing satisfaction across various domains such as physical and mental health, environmental factors, and social aspects (Soresi et al., 2007). Previous research has identified several factors associated with lower quality of life among family caregivers in the realm of cancer care. These factors include being under the age of 35, bearing heavier caregiving responsibilities, having close familial ties with the patient, experiencing low income levels, cohabiting with the patient, lacking sufficient knowledge in managing the patient’s symptoms, and grappling with negative psychological symptoms such as depression and anxiety (Vashistha et al., 2019; Akter et al., 2023; Nayak et al., 2018).

An educational support program tailored for caregivers promotes a triangular relationship among patients, their family caregivers, and healthcare providers, fostering an environment that enhances the quality of life for both patients and caregivers while mitigating caregiver burden (Gabriel et al., 2020; Alfaro-Díaz et al., 2022). Through a comprehensive literature review, numerous interventions have been developed and utilized to enhance quality of life and alleviate caregiver burden among family caregivers of cancer patients (Tan et al., 2018). Interventions within the realm of psycho-oncology are broadly categorized into four main areas: counseling, behavioral methods, physical interventions (Such as aerobics and yoga) (Marshall et al., 2022), and psychosocial interventions (such as psychoeducational support) (Kusi et al., 2023). The latter category may employ various approaches such as cognitive-behavioral techniques, exploratory-interpersonal methods, or psychological and active supportive interventions (Sanjida et al., 2018).

In addressing the challenges faced by caregivers, psychoeducational interventions emerge as a highly beneficial support approach. These interventions employ various methods, including health education, problem-solving skill training, cognitive-behavioral therapy, stress management techniques, coping strategies training, and social support. By imparting knowledge and skills, psychoeducational interventions aim to enhance family caregivers’ understanding of the patient’s illness, bolster their stress-coping abilities, and improve psychological well-being for both caregivers and patients (Barsevick et al., 2002; Sörensen et al., 2002). A psychoeducational support intervention represents a comprehensive, interdisciplinary approach that integrates educational and psychological components. The educational aspect equips caregivers and patients with practical knowledge about cancer, treatment options, potential side effects, complications, and problem-solving strategies. Meanwhile, the psychological component addresses emotional and cognitive aspects of the disease experience, facilitates adaptation to cancer, fosters self-awareness, promotes mood enhancement, teaches stress management techniques, and instills effective problem-solving and coping strategies (Bredal et al., 2014).

In this context, findings from a meta-analysis study have indicated that psychoeducational interventions yield improvements in psychological well-being and coping outcomes among family caregivers of children with cancer. Given the profound impact of coping on both physical and mental health, healthcare professionals may consider integrating coping strategies as a central component within psychoeducational interventions tailored for caregivers of pediatric cancer patients (Tang et al., 2020).

Furthermore, in a study conducted by Chen et al. (2022), results demonstrated the efficacy of psychoeducational interventions in enhancing the quality of life among cancer patients. Extensive research in the field of oncology has consistently shown that following psychoeducational interventions, significant enhancements in quality of life, stress coping skills, and notable reductions in caregiver burden, symptoms of depression, and anxiety have been observed among patients and their family caregivers (Çetin and Nehir, 2020; Çalık et al., 2022; Kusi et al., 2023; Gabriel and Mayers, 2019).

It’s crucial to recognize that enhancing the quality of life and alleviating caregiver burden among family caregivers of cancer patients plays a pivotal role in the patient’s recovery journey. Therefore, the implementation of interventions, such as psychological training, to support these individuals across various educational, psychological, and social dimensions is imperative and essential. Acknowledging the necessity for further research in this domain, this study was conducted with the objective of assessing the efficacy of psychoeducational support interventions on the quality of life and caregiver burden experienced by caregivers of cancer patients. It was hypothesized that such interventions would effectively mitigate caregiver burden and enhance the quality of life.

## 2 Materials and methods

### 2.1 Study design and settings

This non-blinded randomized controlled trial (registered under clinical trial code IRCT20180728040617N3) was conducted in 2024 among family caregivers of cancer patients receiving chemotherapy at the cancer center in Imam Hossein Hospital, Shahroud, Iran.

### 2.2 Participants

**Figure 1:**
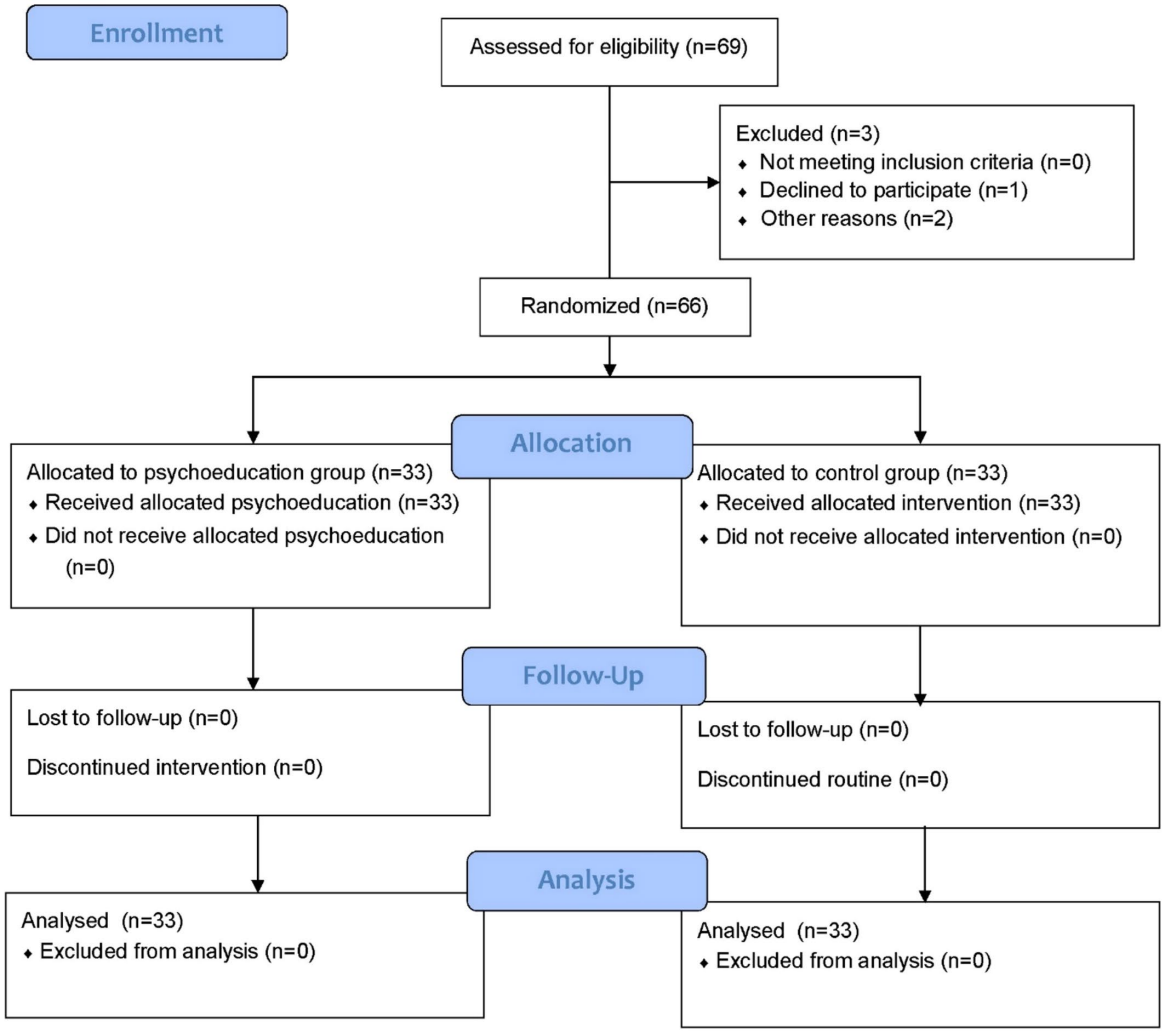
The flow diagram of the study.

Sampling was carried out utilizing a convenience method. Inclusion criteria comprised: (1) age over 18 years, (2) confirmed cancer diagnosis in the patient by an oncologist, (3) undergoing chemotherapy treatment, (4) caregiver possessing Internet and virtual communication capabilities, (5) minimum of 6 months of caregiving experience (Mirhosseini et al., 2024), and (6) possession of a smartphone for communication during the psychoeducation intervention. Exclusion criteria included severe mental disorders, use of neuroleptic drugs, and missing more than two support sessions. Eligible caregivers were informed about the research conditions and procedures via a message sent through virtual social networks such as WhatsApp, inviting them to participate. Two caregivers were excluded from the study due to a lack of smartphone access (Figure 1). The response rate in this study was 97.1%. Allocation in this study was based on random allocation using quadruple block allocation with a ratio of one to one generated by SPSS software.

### 2.3 Intervention

After securing the necessary approvals, the study objectives were initially communicated to all participants, and their informed consent, both verbal and written, was obtained for participation. The intervention sessions were conducted through the WhatsApp social network and were based on Lazarus and Folkman’s Stress, Appraisal, and Coping model (Lazarus and Folkman, 1984). The primary goal of this model was to enhance family caregivers’ ability to manage stressful situations encountered during patient care. This model typically involves four stages:

1. Understanding stress and its implications,
2. Identifying maladaptive thoughts contributing to stress,
3. Substituting these maladaptive thoughts with realistic cognitions and reassessing them, and
4. Employing appropriate coping strategies, encompassing both emotion-oriented and problem-oriented coping, as well as problem-solving skills.

Initially, all participants were briefed on the guidelines for participating in the online groups, emphasizing the importance of sharing only relevant messages pertaining to caregiving experiences and patient needs, while refraining from unrelated content. Each session had a duration ranging from 35 to 45 min. Prior to the sessions, a survey was conducted to determine suitable meeting times, ensuring participants’ availability. They were instructed to be online at the agreed-upon times for active participation. The intervention was administered by the first author, a trained psychiatric nurse, over a period of 3 months, comprising six online group sessions. These sessions encompassed group discussions, question-and-answer sessions, and the exchange of caregiving experiences. The psychoeducational training program in this study was tailored to address the specific context of cancer, its implications, and the unique needs of caregivers of cancer patients undergoing chemotherapy. The psychological strategies underpinning the intervention focused on rectifying maladaptive cognitions, problem-solving techniques, and the effective utilization of coping skills.

**Figure 2:**
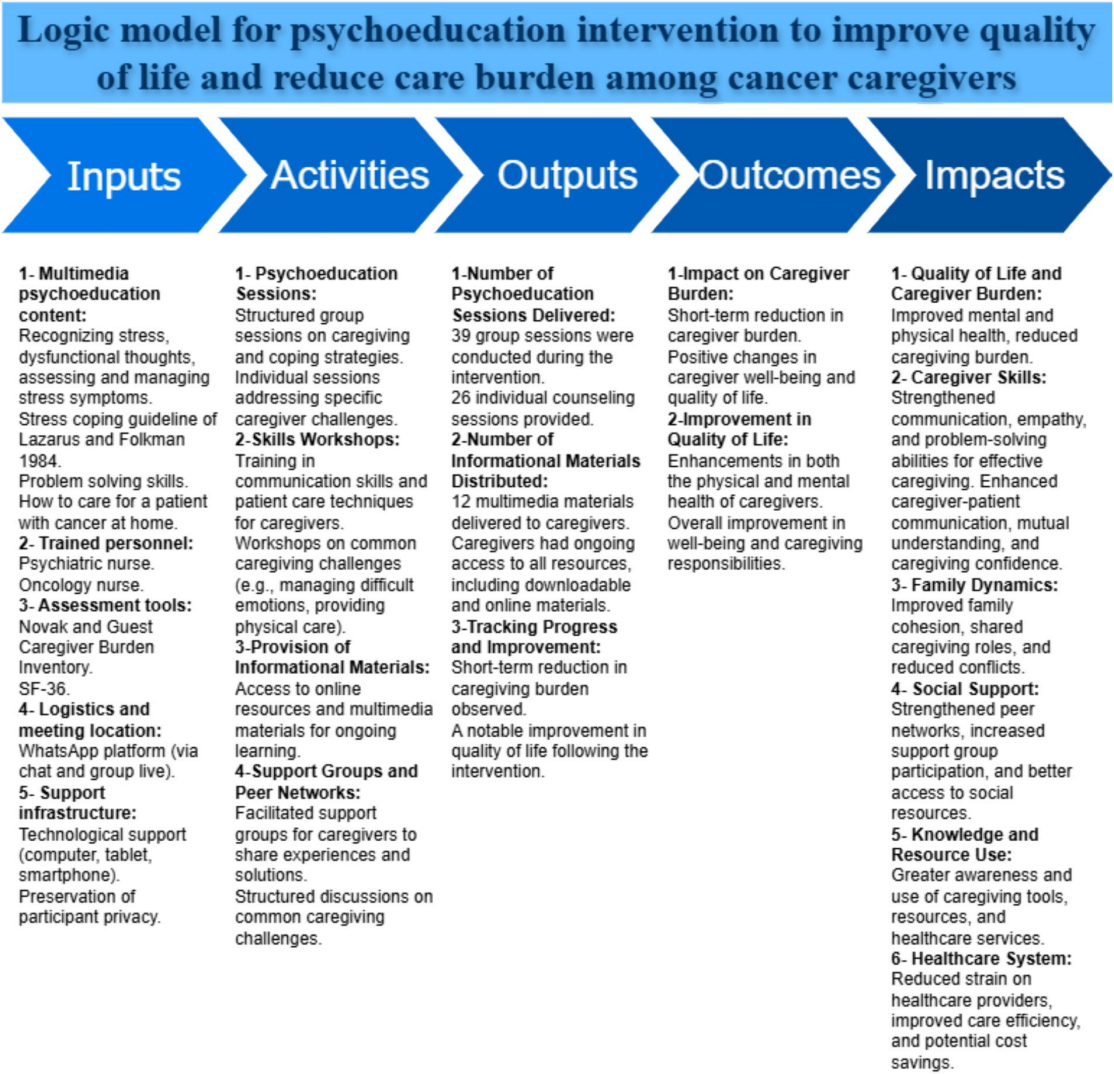
The logic model for the present study.

Additionally, caregivers were encouraged to share their successful caregiving experiences with their patients in the online group chats. Establishing friendly communication among caregivers enabled peer support throughout the intervention. This approach aimed to reduce communication barriers that might exist between nurses and caregivers, allowing caregivers to voice their concerns more openly. Additionally, peers could better understand and relate to the issues being discussed, leading to solutions presented in simpler, more relatable language by fellow caregivers. To facilitate comprehensive learning, educational materials such as appropriate images, slides, and videos were provided alongside practical demonstrations of caregiving techniques. The research team established ongoing communication with the caregivers, ensuring they could reach out for assistance at any time, day or night. Caregivers were provided with a telephone connection to one of the researchers, enabling them to seek support for specific issues or questions as they arose (Figure 2). The above-described interventions adhere to the TIDieR checklist, a tool utilized for organizing details related to behavioral interventions (Hoffmann et al., 2014).

Moreover, participants in the control group received routine interventions provided by medical centers. Following the completion of the study, the aforementioned program was subsequently implemented as a group intervention for the control group.

### 2.4 Instruments

The demographic information form included items such as caregivers’ characteristics (e.g., age, gender, caregiving hours, marital status, education, health insurance, employment status, underlying disease, relationship with the patient, and need for support associations) and some patient-related variables (such as age, duration of illness, gender, and type of cancer), which were assessed in this study.

The study outcomes encompassed caregiving burden and quality of life among caregivers, assessed through self-report questionnaires. Data were collected at baseline and one-month post-intervention.

Quality of life was assessed using the SF-36 questionnaire, which consists of 36 items evaluating eight subscales: physical functioning, role limitations due to physical health, role limitations due to emotional problems, energy/fatigue, emotional well-being, social functioning, pain, and general health. Additionally, it measures the physical and mental components of an individual’s well-being. Higher scores on this questionnaire indicate better quality of life (Ware Jr and Sherbourne, 1992). Previous research has demonstrated that the Persian version of the SF-36 questionnaire exhibits an acceptable minimum reliability coefficient (Montazeri et al., 2005). In the current study, Cronbach’s alpha coefficient was calculated as 0.79 to assess internal consistency.

The Novak and Guest Caregiver Burden Inventory comprises 24 items designed to assess caregiving burden. This questionnaire includes five subscales: time-dependent, developmental, physical, social, and emotional. Caregivers’ responses are rated on a 5-item Likert scale. Scores on this inventory range from a minimum of 24 to a maximum of 120, with caregiving burden categorized as mild (24–47), moderate (48–71), severe (72–95), and very severe (96–120). The inventory demonstrates good reliability, with reported Cronbach’s alpha coefficients for the subscales ranging from 0.69 to 0.87, and an overall Cronbach’s alpha coefficient of 0.80 (Novak and Guest, 1989). In a study by Minaei-Moghadam et al. (2024), the Persian version of this inventory demonstrated a Cronbach’s alpha coefficient of 0.88. In the present study, the reliability of this inventory was assessed as acceptable, with an internal consistency measured by Cronbach’s alpha coefficient calculated at 0.81.

### 2.5 Sample size

The sample size for the present study was determined based on the means and standard deviations reported in previous research for the quality of life and caregiving burden variables (Faridhosseini et al., 2017; Biliunaite et al., 2021). Considering a confidence level of 95% and a power of 80%, and accounting for potential sample dropout, it was estimated that a total of 68 participants (34 individuals in each group) would be needed.

### 2.6 Blinding

In this study, while blinding the participants was not possible given the nature of the intervention, the data collector and statistical consultant maintained blinding throughout the study.

### 2.7 Data analysis

Family caregivers were considered as the primary unit of analysis. Descriptive statistics were used, including frequency and percentage to illustrate variable characteristics (gender, marital status, education, health insurance, employment status, underlying disease, relationship with the patient, type of cancer, and need for support associations), as well as mean and standard deviation for variable distributions (age, caregiving hours, and duration of illness).

The difference between the two groups was assessed using the Chi-square test and Fisher’s exact test. Additionally, the independent t-test was utilized to compare the mean scores of caregiving burden and quality of life between the groups. A significance level of 0.05 was applied to all analyses. Statistical analyses were conducted using the Statistical Package for Social Sciences (SPSS) version 26 and STATA version 17 softwares.

### 2.8 Ethical considerations

Caregivers voluntarily participated in this study after being informed about its objectives and the confidentiality of their information. Data analysis and publication were conducted anonymously and in adherence to ethical standards. Both written and verbal informed consent were obtained from all participants. The study was conducted in accordance with the Declaration of Helsinki and received approval from the Ethics Council of Shahroud University of Medical Sciences (ethics code: IR.SHMU.REC.1399.158). Furthermore, the authors follow the guidelines outlined by the Committee on Publication Ethics (COPE) in disseminating the findings.

## 3 Results

Based on this study’s findings, there were no significant baseline differences between the intervention and control groups regarding demographic variables among caregivers and patients, such as caregiver age, gender, caregiving hours, marital status, education, health insurance, employment, underlying health conditions, relationship to the patient, or the need for support associations. Similarly, patient variables like age, illness duration, gender, and cancer type did not differ significantly between groups (*p* > 0.05). Caregivers in both groups had identical marital status distributions, and all were insured. Gastrointestinal and breast cancers were the most common cancer types, with similar distributions across both groups (*p* = 0.520). Further details are presented in Table 1.

**Table 1:** Demographic information of caregivers of patients with cancer.

Variables		Groups		*p*-value
		Control	Intervention	
		*n* (%)	*n* (%)	
Gender	Male	17 (51.5)	23 (69.7)	0.131*
	Female	16 (48.5)	10 (30.3)	
Level of education	Illiterate	9 (27.3)	9 (27.3)	0.938*
	Secondary school	6 (18.2)	7 (21.2)	
	High school	8 (24.2)	6 (18.2)	
	Academic degree	10 (30.3)	11 (33.3)	
Employment status	Unemployed	9 (27.3)	9 (27.3)	0.122*
	Housewife	11 (33.3)	6 (18.2)	
	Self employed	5 (15.2)	11 (33.3)	
	Retired	3 (9.0)	0 (0.0)	
	Employee	1 (3.0)	5 (15.2)	
	Student	2 (6.1)	1 (3.0)	
	Other	2 (6.1)	1 (3.0)	
Relation	Wife/husband	1 (3.0)	8 (24.2)	0.078*
	Sibling	4 (12.1)	1 (3.0)	
	Parent	10 (30.3)	10 (30.3)	
	Child	16 (48.5)	13 (39.5)	
	Other	2 (6.1)	1 (3.0)	
Underlying disease	Yes	14 (42.4)	9 (27.3)	0.196*
	No	19 (57.6)	24 (72.7)	
Need for support associations	Yes	10 (30.3)	9 (27.3)	0.786*
	No	23 (69.7)	24 (72.7)	
		*Mean ± SD*	*Mean ± SD*	
Caregivers’ age (per year)		43.5 ± 13.6	39.8 ± 9.9	0.208**
Patients’ age (per year)		56.8 ± 10.7	54.3 ± 12.1	0.391**
Duration of illness (per year)		2.1 ± 1.9	1.9 ± 1.5	0.606**
Caregiving hours (per day)		6.1 ± 3.3	7.6 ± 4.0	0.105**

*Chi-squared test; **Independent *t* test; *n*, Frequency; %, Percent; SD, Standard deviation.

Results from the independent t-test indicated that prior to the intervention, there was no significant difference between the two groups in terms of the mean and standard deviation of caregiving burden scores (*p* = 0.980). However, following the intervention, a significant difference emerged between the groups. Specifically, the mean caregiving burden score in the intervention group was significantly lower than that in the control group (*p* = 0.044). Additionally, significant differences were observed between the two groups in terms of the mean changes in caregiving burden scores. Notably, the intervention group exhibited a decrease in caregiving burden scores post-intervention, whereas the control group experienced an increase (2.5 ± 12.0 compared to −4.1 ± 13.7). Further results are detailed in Table 2.

**Table 2:** Mean scores of care burden and its subscales before and after the intervention in both groups.

Variables		Groups		p*
		Control (*n* = 33)	Intervention (*n* = 33)	
		Mean ± SD	Mean ± SD	
Time-dependent	Pre-intervention	13.8 ± 3.4	13.5 ± 3.9	0.787
	Post-intervention	13.5 ± 3.2	12.2 ± 2.5	0.071
	Mean differences	−0.3 ± 3.8	−1.3 ± 3.8	0.260
Developmental	Pre-intervention	13.8 ± 3.7	13.9 ± 4.4	0.904
	Post-intervention	15.3 ± 3.7	13.0 ± 2.6	0.004
	Mean differences	1.5 ± 2.9	−0.9 ± 4.4	0.010
Physical	Pre-intervention	10.1 ± 3.4	10.2 ± 3.7	0.863
	Post-intervention	10.9 ± 2.8	9.2 ± 2.1	0.007
	Mean differences	0.8 ± 3.0	−1.0 ± 3.7	0.031
Social	Pre-intervention	10.4 ± 3.1	10.4 ± 2.9	0.968
	Post-intervention	10.2 ± 4.7	9.3 ± 2.5	0.121
	Mean differences	−0.2 ± 4.0	−1.1 ± 3.3	0.287
Emotional	Pre-intervention	9.6 ± 2.8	9.7 ± 2.6	0.891
	Post-intervention	10.1 ± 2.2	9.9 ± 2.0	0.767
	Mean differences	0.5 ± 2.7	0.2 ± 2.7	0.712
Care burden	Pre-intervention	57.6 ± 13.8	57.7 ± 14.9	0.980
	Post-intervention	60.1 ± 11.1	53.6 ± 9.2	0.013
	Mean differences	2.5 ± 12.0	−4.1 ± 13.7	0.044

*Independent *t* test; *n*, Frequency; *p*, *p*-value; SD, Standard deviation.

As shown in Table 3, no significant differences were observed in the all SF-36 domains and total quality of life scores between the control and psychoeducation groups at baseline (*p* = 0.445). However, post-intervention, the psychoeducation group demonstrated significantly higher mean scores in several SF-36 domains, including role limitations due to emotional problems (*p* = 0.011), energy/fatigue (*p* = 0.002), emotional well-being (*p* = 0.001), social functioning (*p* = 0.021), and pain (*p* = 0.015) compared to the control group. Overall, the psychoeducation group had significantly higher quality of life scores than the control group (p = 0.001). The intervention group demonstrated an increase in average quality of life scores (4.7 ± 16.9), while the control group showed a decline (−8.6 ± 15.3), highlighting a substantial difference between the groups. Using STATA software, a power analysis was conducted for the main variables, care burden and quality of life, revealing a power of 1.0 for both.

**Table 3:** Quality of life and its dimensions before and after the intervention.

Variables		Groups		p*
		Control (*n* = 33)	Intervention (*n* = 33)	
		Mean ± SD	Mean ± SD	
Physical functioning	Pre-intervention	80.6 ± 29.0	81.1 ± 21.5	0.943
	Post-intervention	72.7 ± 25.1	85.0 ± 14.2	0.028
	Mean differences	−7.9 ± 24.9	3.0 ± 24.1	0.076
Role limitations due to physical health	Pre-intervention	76.5 ± 38.5	71.2 ± 32.5	0.548
	Post-intervention	72.7 ± 28.9	80.3 ± 25.6	0.264
	Mean differences	−3.8 ± 33.7	9.1 ± 37.4	0.147
Role limitations due to emotional problems	Pre-intervention	60.6 ± 40.4	53.5 ± 41.6	0.486
	Post-intervention	40.4 ± 36.1	61.6 ± 27.8	0.009
	Mean differences	−20.2 ± 44.8	8.1 ± 42.6	0.011
Energy/fatigue	Pre-intervention	57.0 ± 15.6	51.8 ± 16.1	0.192
	Post-intervention	50.2 ± 11.6	57.1 ± 10.1	0.011
	Mean differences	−6.8 ± 14.7	5.3 ± 16.2	0.002
Emotional well-being	Pre-intervention	58.2 ± 13.9	54.3 ± 14.6	0.274
	Post-intervention	50.9 ± 11.9	58.3 ± 11.1	0.011
	Mean differences	−7.3 ± 11.6	4.0 ± 15.1	0.001
Social functioning	Pre-intervention	68.6 ± 17.7	65.9 ± 17.2	0.539
	Post-intervention	60.2 ± 12.3	67.0 ± 12.8	0.031
	Mean differences	−8.3 ± 12.4	1.1 ± 19.1	0.021
Pain	Pre-intervention	83.9 ± 20.1	79.1 ± 24.3	0.388
	Post-intervention	73.6 ± 21.2	84.3 ± 18.3	0.032
	Mean differences	−10.3 ± 23.4	5.2 ± 26.0	0.015
General health	Pre-intervention	49.8 ± 17.7	51.1 ± 16.3	0.773
	Post-intervention	45.6 ± 12.4	52.9 ± 12.4	0.020
	Mean differences	−4.2 ± 14.8	1.8 ± 15.4	0.108
Quality of life	Pre-intervention	66.9 ± 18.8	63.5 ± 17.0	0.445
	Post-intervention	58.3 ± 13.9	68.2 ± 11.1	0.002
	Mean differences	−8.6 ± 15.3	4.7 ± 16.9	0.001

*Independent *t* test; *n*, Frequency; *p*, *p*-value; SD, Standard deviation.

Moreover, linear regression analysis revealed that the psychoeducation group had significantly lower caregiving burden scores and higher quality of life scores post-intervention compared to the control group. However, the time × group interaction effect was not significant for either caregiving burden or quality of life (Table 4).

**Table 4:** Effect of psychoeducation intervention on quality of life and care burden by regression analysis.

Variables			β	SE	*t*	*p*-value
Quality of life	Constant value		46.573	5.269	8.839	<0.001
	Time		0.341	0.077	4.412	<0.001
	Group	Psychoeducation	Ref			
		Control	−11.068	2.739	−4.041	<0.001
	Time × group		−0.224	0.154	−1.457	0.150
Care burden	Constant value		33.389	4.780	6.986	<0.001
	Time		0.351	0.078	4.482	<0.001
	Group	Psychoeducation	Ref			
		Control	6.456	2.210	2.922	0.005
	Time × group		−0.174	0.157	−1.110	0.271

SE, Standard error.

## 4 Discussion

The current study aimed to assess the impact of psychoeducation intervention on caregiving burden and quality of life among caregivers of cancer patients. The findings of this research indicate that implementing psychoeducation intervention, which focuses on enhancing caregivers’ coping skills, problem-solving abilities, and stress management techniques, may likely enhance quality of life and alleviate caregiving burden.

In the process of coping, utilizing coping skills and problem-solving techniques is generally considered beneficial for managing stress. Coping entails how individuals respond and behave when faced with stress, especially during heightened levels of stress exposure. Coping strategies refer to cognitive and behavioral efforts individuals employ to interpret and overcome challenges and difficulties (Lazarus and Folkman, 1984). Caregivers of cancer patients encounter various stressors, highlighting the importance of effectively utilizing these skills. Previous studies have established a significant correlation between levels of caregiving burden and coping strategies. Problem-based coping has been linked to reduced levels of caregiving burden, depressive symptoms, and better adaptation, whereas emotion-based coping has been associated with post-traumatic growth among caregivers of cancer patients (Akpan-Idiok et al., 2020; Teixeira et al., 2018). Thus, it is hypothesized that following the implementation of psychoeducation intervention, caregivers will experience a reduction in caregiving burden while effectively employing coping strategies, problem-solving skills, and appropriate stress management techniques. Supporting this hypothesis, a systematic review and meta-analysis have demonstrated the effectiveness of psychoeducation-based interventions in alleviating negative psychological effects and enhancing coping skills among caregivers of children with cancer (Phiri et al., 2023).

Based on the results of the current study, it was observed that following the implementation of psychoeducation intervention, there was a significant decrease in caregiving burden scores. This aligns with the findings of a systematic review and meta-analysis conducted by Cheng et al. (2022), which demonstrated the efficacy of psychoeducational interventions in alleviating symptoms of anxiety, depression, and caregiving burden, while enhancing self-efficacy and quality of life among caregivers of cancer patients. Additionally, previous research has highlighted the beneficial impact of psychoeducational interventions on quality of life, caregiving burden, and the reduction of anxiety and depression symptoms in caregivers of cancer patients (Kusi et al., 2023). Furthermore, the effectiveness of such interventions in reducing caregiving burden has been demonstrated in other contexts. For instance, a study by Hemmati Maslakpak et al. (2019) illustrated that psychoeducation intervention, comprising six group discussion sessions and four workshop sessions, significantly reduced the caregiving burden among caregivers of hemodialysis patients. Despite variations in the implementation of psychoeducation intervention across studies, the overarching findings remain consistent. These findings extend beyond cancer caregiving to other chronic diseases such as schizophrenia, dementia, multiple sclerosis, and heart failure, thereby corroborating the results of the current study (Frias et al., 2020; Okafor and Monahan, 2023; Douglas et al., 2021; Cassidy et al., 2021).

It’s essential to recognize that changes in caregiving burden play a significant role in predicting caregivers’ quality of life within the realm of cancer care. Improved quality of life is often associated with a reduction in caregiving burden (Akter et al., 2023). Therefore, in line with the second hypothesis of this study, it is anticipated that quality of life will enhance following the implementation of the intervention, concurrent with the observed reduction in caregiving burden. This assertion is supported by findings from a study conducted in Singapore, where a brief weekly psychoeducation intervention over 4 weeks notably enhanced the quality of life of caregivers of cancer patients (Tan et al., 2018). Similarly, Gabriel et al. demonstrated the effectiveness of a psychoeducation intervention comprising six weekly 90-min sessions in enhancing the quality of life of caregivers of breast cancer patients, aligning with the present study’s outcomes (Gabriel and Mayers, 2019). Furthermore, results from a similar study conducted in Turkey echoed the current findings, showcasing improvements in quality of life for both patients with incurable cancer and their caregivers (Çalık et al., 2022).

Some studies, including the current one, have implemented psychoeducation-based support online. Utilizing remote support interventions offers several advantages. Caregivers often find it challenging to be physically present at the patient’s bedside and may incur expenses to participate in face-to-face intervention sessions. Similarly, in alignment with the findings of the current study, a systematic review and meta-analysis revealed that online health interventions, such as online psychoeducation, are effective in supporting informal caregivers of cancer patients, leading to a reduction in depressive symptoms and an improvement in their quality of life (Li et al., 2022). Research conducted in other domains, such as bipolar disorder, chronic kidney disease, patients receiving palliative care, and older adults with mild cognitive impairment, has also yielded similar results (Bártolo et al., 2022; Baruch et al., 2018; Çetin and Nehir, 2020; Young et al., 2019).

Another feature of the intervention employed in the current study was its group-based implementation. Psychoeducation interventions can be conducted either individually or in a group setting. In the group approach, caregivers have the opportunity to exchange their caregiving experiences with one another. From the authors’ perspective, this aspect represents an added advantage of the psychoeducational intervention. By engaging in direct communication with fellow caregivers who share similar circumstances, individuals gain a deeper understanding of the emotions, experiences, and challenges associated with caregiving (Bilenchi et al., 2022; Khouban-Shargh et al., 2024).

Previous research has shown variations in disease types, psychoeducation intervention models, and socio-cultural contexts. Nevertheless, their collective outcomes consistently underscore the beneficial effectiveness of such interventions in mitigating negative outcomes and fostering positive ones in caregiving. Given that this study was conducted within Iranian society, which harbors distinct religious and social beliefs, caution is warranted when extrapolating the findings to other cultures and societies. Furthermore, data collection for this research relied on questionnaires and scales, thereby posing a threat to the external validity of the results due to potential response bias. Moreover, since the data collection tools were not specifically tailored for the cancer context, the results are susceptible to measurement error. Additionally, the effectiveness of the intervention was not assessed over multiple or long-term periods. Thus, it’s pertinent to mention that its impact should be evaluated at different time stages in future studies. Although it is well established that greater disease severity is associated with higher caregiving burden and lower quality of life, we were unable to adequately measure this variable due to insufficient information on disease stage in patients’ medical records. We recommend that future studies address this gap. The convenience sampling method offers greater flexibility in participant recruitment. However, it is important to note that this approach can introduce selection bias into the study results. Future studies are advised to take this limitation into consideration. Despite these limitations, it’s essential to underscore the novelty of this study in integrating Lazarus and Folkman’s Stress, Appraisal, and Coping model into the cancer domain and assessing its effects on various caregiving aspects, particularly caregiving burden and quality of life among family caregivers. These findings hold significant implications for the clinical implementation of similar support programs.

### 4.1 Implications for practice

This study underscores the short-term need to incorporate psychoeducational interventions into routine care for caregivers of cancer patients. These programs have been shown to significantly reduce caregiving burden and enhance quality of life by fostering coping skills, stress management, and problem-solving abilities. To maximize accessibility and cost-effectiveness, early implementation of group-based and online delivery models is highly recommended, particularly to address common challenges faced by caregivers in Iran.

Cultural and contextual adaptations are vital to ensure these interventions are relevant and effective across diverse populations. Policymakers and practitioners are encouraged to develop standardized, culturally sensitive protocols for integrating psychoeducation into healthcare systems. Additionally, clinicians should receive training to seamlessly incorporate these programs into their routine practice.

To sustain the benefits of psychoeducation, follow-up strategies such as booster sessions and continuous access to supportive resources are crucial. This study advocates for the development of clinical guidelines and budgetary policies that prioritize psychoeducational interventions, ultimately enhancing caregiver well-being and improving the quality of care for patients.

## 5 Conclusion

Family caregivers of cancer patients frequently encounter a decline in their quality of life and grapple with substantial caregiving burdens. Implementing psychoeducational support aimed at enhancing coping skills, problem-solving abilities, and stress management in caregivers is recommended as an effective, practical, and cost-efficient intervention to alleviate caregiving burdens and enhance their quality of life.

## Data Availability

The raw data supporting the conclusions of this article will be made available by the authors, without undue reservation.

## References

[ref1] Adelman R. D. Tmanova L. L. Delgado D. Dion S. Lachs M. S. (2014). Caregiver burden: a clinical review. JAMA 311, 1052–1060. doi: 10.1001/jama.2014.304, PMID: 24618967

[ref2] Akpan-Idiok P. A. Ehiemere I. O. Asuquo E. F. Chabo J. A. U. Osuchukwu E. C. (2020). Assessment of burden and coping strategies among caregivers of cancer patients in sub-Saharan Africa. World J. Clin. Oncol. 11, 1045–1063. doi: 10.5306/wjco.v11.i12.1045, PMID: 33437666 PMC7769710

[ref3] Akter J. Konlan K. D. Nesa M. Ispriantari A. (2023). Factors influencing cancer patients’ caregivers’ burden and quality of life: an integrative review. Heliyon 9:e21243. doi: 10.1016/j.heliyon.2023.e21243, PMID: 38027739 PMC10643105

[ref4] Alfaro-Díaz C. Svavarsdottir E. K. Esandi N. Klinke M. E. Canga-Armayor A. (2022). Effectiveness of nursing interventions for patients with cancer and their family members: a systematic review. J. Fam. Nurs. 28, 95–114. doi: 10.1177/10748407211068816, PMID: 35057657

[ref5] Barsevick A. M. Sweeney C. Haney E. Chung E. (2002). A systematic qualitative analysis of psychoeducational interventions for depression in patients with cancer. Oncol. Nurs. Forum 29, 73–84. doi: 10.1188/02.ONF.73-87, PMID: 11817494

[ref6] Bártolo A. Sousa H. Ribeiro O. Figueiredo D. (2022). Effectiveness of psychosocial interventions on the burden and quality of life of informal caregivers of hemodialysis patients: a systematic review. Disabil. Rehabil. 44, 8176–8187. doi: 10.1080/09638288.2021.2013961, PMID: 34913777

[ref7] Baruch E. Pistrang N. Barker C. (2018). Psychological interventions for caregivers of people with bipolar disorder: a systematic review and meta-analysis. J. Affect. Disord. 236, 187–198. doi: 10.1016/j.jad.2018.04.077, PMID: 29747136

[ref8] Bilenchi V. A. Banfi P. Pagnini F. Volpato E. (2022). Psychoeducational groups for people with amyotrophic lateral sclerosis and their caregiver: a qualitative study. Neurol. Sci. 43, 4239–4255. doi: 10.1007/s10072-022-05930-2, PMID: 35156152

[ref9] Biliunaite I. Kazlauskas E. Sanderman R. Truskauskaite-Kuneviciene I. Dumarkaite A. Andersson G. (2021). Internet-based cognitive behavioral therapy for informal caregivers: randomized controlled pilot trial. J. Med. Internet Res. 23:e21466. doi: 10.2196/21466, PMID: 33825687 PMC8060860

[ref10] Bredal I. S. Kåresen R. Smeby N. A. Espe R. Sørensen E. M. Amundsen M.. (2014). Effects of a psychoeducational versus a support group intervention in patients with early-stage breast cancer: results of a randomized controlled trial. Cancer Nurs. 37, 198–207. doi: 10.1097/NCC.0b013e31829879a3, PMID: 23782517

[ref11] Çalık K. Y. Küçük E. Halimoğlu B. (2022). The effect of an educational palliative care intervention on the quality of life of patients with incurable cancer and their caregivers. Supportive Care Cancer 30, 2427–2434. doi: 10.1007/s00520-021-06672-1 34761298

[ref12] Caserta M. S. Lund D. A. Wright S. D. (1996). Exploring the caregiver burden inventory (CBI): further evidence for a multidimensional view of burden. Int. J. Aging Hum. Dev. 43, 21–34. doi: 10.2190/2DKF-292P-A53W-W0A8, PMID: 8886874

[ref13] Cassidy L. Hill L. Fitzsimons D. McGaughey J. (2021). The impact of psychoeducational interventions on the outcomes of caregivers of patients with heart failure: a systematic review and meta-analysis. Int. J. Nurs. Stud. 114:103806. doi: 10.1016/j.ijnurstu.2020.103806, PMID: 33248290

[ref14] Çetin Ö. Nehir S. (2020). Effects of psychoeducation on palliative caregivers’ quality of life and skills to cope with stress. Cukurova Med. J. 45, 785–794. doi: 10.17826/cumj.649713

[ref15] Chen D. Liu Q. Zhang L. Qian H. (2022). Effectiveness of dyadic psychoeducational intervention on cancer patients and their caregivers: a systematic review and meta-analysis. Cancer Nurs. 10:1097. doi: 10.1097/NCC.0000000000001307, PMID: 38011076

[ref16] Cheng Q. Binbin X. Ng M. S. N. Duan Y. So W. K. W. (2022). Effectiveness of psychoeducational interventions among caregivers of patients with cancer: a systematic review and meta-analysis. Int. J. Nurs. Stud. 127:104162. doi: 10.1016/j.ijnurstu.2021.104162, PMID: 35121521

[ref17] Dengsø K. E. Thomsen T. Andersen E. W. Hansen C. P. Christensen B. M. Hillingsø J.. (2021). The psychological symptom burden in partners of pancreatic cancer patients: a population-based cohort study. Support. Care Cancer 29, 6689–6699. doi: 10.1007/s00520-021-06251-4, PMID: 33963908

[ref18] Douglas S. L. Plow M. Packer T. Lipson A. R. Lehman M. J. (2021). Psychoeducational interventions for caregivers of persons with multiple sclerosis: protocol for a randomized trial. JMIR Res. Protoc. 10:e30617. doi: 10.2196/30617, PMID: 34435971 PMC8430872

[ref19] Falconier M. K. Jackson J. B. Hilpert P. Bodenmann G. (2015). Dyadic coping and relationship satisfaction: a meta-analysis. Clin. Psychol. Rev. 42, 28–46. doi: 10.1016/j.cpr.2015.07.002, PMID: 26295276

[ref20] Faridhosseini F. Baniasadi M. Bordbar M. R. F. Pourgholami M. Ahrari S. Asgharipour N. (2017). Effectiveness of psychoeducational group training on quality of life and recurrence of patients with bipolar disorder. Iran. J. Psychiatry 12, 21–28, PMID: 28496498 PMC5425348

[ref21] Frias C. E. Garcia-Pascual M. Montoro M. Ribas N. Risco E. Zabalegui A. (2020). Effectiveness of a psychoeducational intervention for caregivers of people with dementia with regard to burden, anxiety and depression: a systematic review. J. Adv. Nurs. 76, 787–802. doi: 10.1111/jan.14286 31808211

[ref22] Fumaneeshoat O. Ingviya T. (2020). Quality of life and burden of lung cancer patients’ caregivers: a cross-sectional study from southern Thailand. J. Health Sci. Med. Res. 38, 177–192. doi: 10.31584/jhsmr.2020736

[ref23] Gabriel I. Creedy D. Coyne E. (2020). A systematic review of psychosocial interventions to improve quality of life of people with cancer and their family caregivers. Nurs. Open 7, 1299–1312. doi: 10.1002/nop2.543, PMID: 32802350 PMC7424465

[ref24] Gabriel I. O. Mayers P. M. (2019). Effects of a psychosocial intervention on the quality of life of primary caregivers of women with breast cancer. Eur. J. Oncol. Nurs. 38, 85–91. doi: 10.1016/j.ejon.2018.12.003, PMID: 30717942

[ref25] Goyanka R. (2021). Economic and non-economic burden of cancer: a propensity score matched analysis using household health survey data of India. Cancer Res. Stat. Treat. 4, 29–36. doi: 10.4103/crst.crst_6_21

[ref26] Harding R. (2013). Informal caregivers in home palliative care. Prog. Palliat. Care 21, 229–231. doi: 10.1179/1743291X13Y.0000000056

[ref27] Hastert T. A. Ruterbusch J. J. Nair M. Noor M. I. Beebe-Dimmer J. L. Schwartz K.. (2020). Employment outcomes, financial burden, anxiety, and depression among caregivers of African American cancer survivors. JCO Oncol. Pract. 16, e221–e233. doi: 10.1200/JOP.19.00410, PMID: 31496392 PMC7069702

[ref28] Herschbach P. Britzelmeir I. Dinkel A. Giesler J. M. Herkommer K. Nest A.. (2020). Distress in cancer patients: who are the main groups at risk? Psycho-Oncology 29, 703–710. doi: 10.1002/pon.5321, PMID: 31876011

[ref29] Ho P. J. Gernaat S. A. M. Hartman M. Verkooijen H. M. (2018). Health-related quality of life in Asian patients with breast cancer: a systematic review. BMJ Open 8:e020512. doi: 10.1136/bmjopen-2017-020512, PMID: 29678980 PMC5914715

[ref30] Hoffmann T. C. Glasziou P. P. Boutron I. Milne R. Perera R. Moher D.. (2014). Better reporting of interventions: template for intervention description and replication (TIDieR) checklist and guide. Br. Med. J. 348:g1687. doi: 10.1136/bmj.g1687, PMID: 24609605

[ref31] Kayser K. Watson L. E. Andrade J. T. (2007). Cancer as a" we-disease": examining the process of coping from a relational perspective. Fam. Syst. Health 25, 404–418. doi: 10.1037/1091-7527.25.4.404

[ref32] Khouban-Shargh R. Mirhosseini S. Ghasempour S. Basirinezhad M. H. Abbasi A. (2024). Stress management training program to address caregiver burden and perceived stress among family caregivers of patients undergoing hemodialysis: a randomized controlled trial study. BMC Nephrol. 25:350. doi: 10.1186/s12882-024-03795-5, PMID: 39402480 PMC11476389

[ref33] Kilic S. T. Oz F. (2019). Family caregivers’ involvement in caring with cancer and their quality of life. Asian Pac. J. Cancer Prev. 20, 1735–1741. doi: 10.31557/APJCP.2019.20.6.1735, PMID: 31244294 PMC7021632

[ref34] Kusi G. Atenafu E. G. Mensah A. B. B. Lee C. T. Viswabandya A. Puts M.. (2023). The effectiveness of psychoeducational interventions on caregiver-oriented outcomes in caregivers of adult cancer patients: a systematic review and meta-analysis. Psycho-Oncology 32, 189–202. doi: 10.1002/pon.6050, PMID: 36251609

[ref35] Lazarus R. S. Folkman S. (1984). Stress, appraisal, and coping. New York: Springer Publishing Company.

[ref36] Lewandowska A. Rudzki G. Lewandowski T. Rudzki S. (2021). The problems and needs of patients diagnosed with cancer and their caregivers. Int. J. Environ. Res. Public Health 18:87. doi: 10.3390/ijerph18010087, PMID: 33374440 PMC7795845

[ref37] Li Y. Li J. Zhang Y. Ding Y. Xiaolin H. (2022). The effectiveness of e-health interventions on caregiver burden, depression, and quality of life in informal caregivers of patients with cancer: a systematic review and meta-analysis of randomized controlled trials. Int. J. Nurs. Stud. 127:104179. doi: 10.1016/j.ijnurstu.2022.104179, PMID: 35124473

[ref38] Marshall E. LaCaille R. A. LaCaille L. J. Lee J. E. Peterson E. (2022). Effects of physical activity interventions for caregivers of adults: a meta-analysis. Health Psychol. 41, 585–598. doi: 10.1037/hea0001212, PMID: 35797152

[ref39] Maslakpak H. Masumeh M. T. Radfar M. Alinejad V. (2019). The effect of psycho-educational intervention on the caregiver burden among caregivers of hemodialysis patients. J. Res. Dev. Nurs. Midw. 16, 14–25. doi: 10.29252/jgbfnm.16.1.14

[ref40] Minaei-Moghadam S. Manzari Z. S. Vaghee S. Mirhosseini S. (2024). Effectiveness of a supportive care program via a smartphone application on the quality of life and care burden among family caregivers of patients with major depressive disorder: a randomized controlled trial. BMC Public Health 24:66. doi: 10.1186/s12889-023-17594-4, PMID: 38166907 PMC10762964

[ref41] Mirhosseini S. Nezhad F. S. H. Rahim A. H. M. Basirinezhad M. H. Bakhshiarab A. Saeedi M.. (2024). Care burden and the predictive role of spiritual well-being and religious coping: a cross sectional study among Iranian family caregivers of patients with stroke. Health Sci. Rep. 7:e2155. doi: 10.1002/hsr2.2155, PMID: 38841117 PMC11150275

[ref42] Montazeri A. Goshtasebi A. Vahdaninia M. Gandek B. (2005). The short form health survey (SF-36): translation and validation study of the Iranian version. Qual. Life Res. 14, 875–882. doi: 10.1007/s11136-004-1014-5, PMID: 16022079

[ref43] Nayak M. G. George A. Vidyasagar M. S. (2018). Perceived barriers to symptoms management among family caregivers of cancer patients. Indian J. Palliat. Care 24, 202–206. doi: 10.4103/IJPC.IJPC_27_18, PMID: 29736126 PMC5915890

[ref44] Novak M. Guest C. (1989). Application of a multidimensional caregiver burden inventory. The Gerontologist 29, 798–803. doi: 10.1093/geront/29.6.798, PMID: 2516000

[ref45] Ochoa C. Y. Lunsford N. B. Smith J. L. (2020). Impact of informal cancer caregiving across the cancer experience: a systematic literature review of quality of life. Palliat. Support. Care 18, 220–240. doi: 10.1017/S1478951519000622, PMID: 31588882 PMC8678891

[ref46] Okafor A. J. Monahan M. (2023). Effectiveness of psychoeducation on burden among family caregivers of adults with schizophrenia: a systematic review and meta-analysis. Nurs. Res. Pract. 2023, 2167096–2167016. doi: 10.1155/2023/2167096, PMID: 37767330 PMC10522442

[ref47] Phiri L. Li W. H. C. Cheung A. T. Phiri P. G. M. C. (2023). Effectiveness of psychoeducation interventions in reducing negative psychological outcomes and improving coping skills in caregivers of children with cancer: a systematic review and meta-analysis. Psycho-Oncology 32, 1514–1527. doi: 10.1002/pon.6208, PMID: 37639282

[ref48] Sanjida S. McPhail S. M. Shaw J. Couper J. Kissane D. Price M. A.. (2018). Are psychological interventions effective on anxiety in cancer patients? A systematic review and meta-analyses. Psycho-Oncology 27, 2063–2076. doi: 10.1002/pon.4794, PMID: 29885258

[ref49] Sörensen S. Pinquart M. Duberstein P. (2002). How effective are interventions with caregivers? An updated meta-analysis. The Gerontologist 42, 356–372. doi: 10.1093/geront/42.3.356, PMID: 12040138

[ref50] Soresi S. Nota L. Ferrari L. (2007). Considerations on supports that can increase the quality of life of parents of children with disabilities. J. Policy Pract. Intellect. Disabil. 4, 248–251. doi: 10.1111/j.1741-1130.2006.00087.x

[ref51] Tan J. Y. S. Lam K. F. Y. Lim H. A. Chua S. M. Kua E. H. Griva K.. (2018). Post-intervention sustainability of a brief psycho-educational support group intervention for family caregivers of cancer patients. Asia Pac. Psychiatry 10:e12305. doi: 10.1111/appy.12305, PMID: 29226634

[ref52] Tang W. P. Y. Chan C. W. H. Leung D. Y. P. Chan D. N. S. (2020). The effects of psychoeducational interventions on caregivers of children with cancer: a meta-analysis of randomized controlled trials. J. Child Health Care 24, 123–142. doi: 10.1177/1367493518814917, PMID: 30654630

[ref53] Teixeira R. J. Applebaum A. J. Bhatia S. Brandão T. (2018). The impact of coping strategies of cancer caregivers on psychophysiological outcomes: an integrative review. Psychol. Res. Behav. Manag. 11, 207–215. doi: 10.2147/PRBM.S164946, PMID: 29872357 PMC5973462

[ref54] Turliuc M. N. Milek A. Trillingsgaard T. (2021). Individual versus dyadic processes: Health and relationship outcomes. Front. Psychol. 12:714548. doi: 10.3389/fpsyg.2021.714548 34367035 PMC8337046

[ref55] Üzar-Özçetin Y. S. Dursun S. İ. (2020). Quality of life, caregiver burden, and resilience among the family caregivers of cancer survivors. Eur. J. Oncol. Nurs. 48:101832. doi: 10.1016/j.ejon.2020.101832 32949940

[ref56] Vashistha V. Poulose R. Choudhari C. Kaur S. Mohan A. (2019). Quality of life among caregivers of lower-income cancer patients: a single-institutional experience in India and comprehensive literature review. Asian Pac. J. Cancer Care 4, 87–93. doi: 10.31557/apjcc.2019.4.3.87-93

[ref57] Vatter S. McDonald K. R. Stanmore E. Clare L. Leroi I. (2018). Multidimensional care burden in Parkinson-related dementia. J. Geriatr. Psychiatry Neurol. 31, 319–328. doi: 10.1177/0891988718802104, PMID: 30244631

[ref58] Ware J. E. Jr. Sherbourne C. D. (1992). The MOS 36-item short-form health survey (SF-36): I. Conceptual framework and item selection. Med. Care 30, 473–483. doi: 10.1097/00005650-199206000-00002, PMID: 1593914

[ref59] Young D. K.-w. Ng P. Y.-n. Cheng D. (2019). Psychoeducation group on improving quality of life of mild cognitive impaired elderly. Res. Soc. Work. Pract. 29, 303–310. doi: 10.1177/1049731517732420

